# Psychosocial stress and risk of first-ever stroke: a systematic review and meta-analysis

**DOI:** 10.1186/s12883-026-05307-4

**Published:** 2026-09-23

**Authors:** Mostafa A. Khalifa, Diaaeldin Hassanein, Abd Alrahman I. Alsabbagh, Ahmed Samir Gamil, Darin Emad Abdelhaleem, Ayham R. A. Zidat, Eman Atef Megahed, Malak A. Ghazi, Mahmoud Hamza Abdelfattah, Moustafa Mahmoud Aboutabl, Osama A. Ibrahim, Abrar M. Adil, Amal F. Ahmed, Hassan Ahmed AbdelKareem, Mohamed Mounir Gharib, Mustafa H. Khazali

**Affiliations:** 1 Faculty of Medicine, Cairo University, Cairo, Egypt; 2 Faculty of Medicine, King Salman International University, El-Tur, South Sinai, Egypt; 3 Faculty of Medicine, Ain Shams University, Cairo, Egypt; 4 Faculty of Pharmacy, Tanta University, Tanta, Egypt; 5 Faculty of Medicine, Palestine Polytechnic University, Hebron, Palestine; 6 El-Mahalla Ophthalmology Hospital, El-Mahalla El-Kubra, Gharbeya, Egypt; 7 Faculty of Medicine, Alexandria University, Alexandria, Egypt; 8 Faculty of Medicine, Granada University, Granada, Spain; 9 Faculty of Medicine, Alexandria National University, Alexandria, Egypt; 10 Faculty of Medicine, Ahfad University for Women, Omdurman, Sudan; 11 Faculty of Medicine, Ahfad University for Women, Omdurman, Sudan; 12 Faculty of Medicine, Al Azhar Cairo University of Medicine, Cairo, Egypt; 13 Faculty of Pharmacy, Zagazig University, El Sharkia, Egypt; 14 University of Baghdad College of Medicine, Baghdad, Iraq

**Keywords:** Stroke, Stress, Psychological, Occupational Stress, Cerebrovascular Disorders, Meta-Analysis

## Abstract

**Background:**

Psychosocial stress has been recognized as a potential risk factor for stroke; however, the strength and consistency of its association with first-ever stroke remain uncertain. This systematic review and meta-analysis aimed to determine the overall association between psychosocial stress and the risk of first stroke in a stroke-free population.

**Methods:**

We conducted a systematic review and meta-analysis in accordance with the PRISMA and MOOSE guidelines. PubMed, Scopus, Web of Science, and Ovid were searched from inception to 1 January 2026 for cohort or case–control studies. Eligible studies enrolled participants without a prior history of stroke and reported adjusted risk estimates with 95% confidence intervals. Psychosocial stressors were categorized into psychological, vocational, interpersonal, and behavioral domains. Stroke outcomes included ischemic stroke, hemorrhagic stroke, subarachnoid hemorrhage, transient ischemic attack, and unspecified stroke.

**Results:**

Eighty-four studies were included (72 prospective cohort and 12 case–control), comprising 4,128,167 participants. In cohort studies, psychological stress was associated with a higher risk of first-ever stroke (hazard ratio [HR] 1.27, 95% confidence interval 1.19–1.36). Vocational stressors were also associated with increased stroke risk (HR 1.32, 95% CI 1.23–1.43). Interpersonal stressors showed a smaller but statistically significant association (HR 1.11, 95% CI 1.03–1.20). Behavioral stress-related traits showed the largest effect estimate (HR 1.51, 95% CI 1.05–2.15), although this finding should be interpreted cautiously because it was based on only three cohort studies. In sex-stratified analyses, the association was significant among men (HR 1.19, 95% CI 1.07–1.32) but not among women (HR 0.86, 95% CI 0.58–1.28); however, the subgroup interaction was not statistically significant and should be interpreted as inconclusive rather than evidence of a definite sex-specific difference.

**Conclusion:**

Psychosocial stress is associated with an increased risk of first-ever stroke, particularly psychological and vocational stressors, supporting psychosocial factors as potentially modifiable components of stroke prevention.

**Supplementary Information:**

The online version contains supplementary material available at https://doi.org/10.1186/s12883-026-05307-4.

## Introduction

According to the World Health Organization, stroke is defined as “rapidly developed clinical signs of focal or global disturbance of cerebral function, lasting more than 24 h or leading to death, with no apparent cause other than vascular origin” [1]. Stroke is one of the leading causes of death and long-term disability worldwide, placing a substantial and growing burden on healthcare systems [2]. In 2021, an estimated 11.9 million individuals experienced a stroke [3]. Projections indicate that stroke-related mortality may increase by nearly 50% between 2020 and 2050 [2], highlighting the urgent need to better understand both established and emerging modifiable risk factors [3].

While hypertension, diabetes, and high cholesterol are well-established contributors to stroke [4], psychosocial factors—encompassing psychological distress, behavioral, interpersonal, and vocational domains—are less clearly understood and require further investigation to clarify their role [5]. Since most strokes are preventable [6], identifying and addressing modifiable risk factors remain a major public health priority. Evidence from large international studies indicates that a limited set of vascular, lifestyle, and psychosocial determinants accounts for nearly 90% of the population-attributable risk of stroke [7], emphasizing the importance of prevention strategies [8].

In this context, psychosocial factors have emerged as a potentially important contributor to stroke risk [9]. Early systematic reviews and meta-analyses suggested an association between psychosocial stress and stroke, reporting a 30–45% higher risk among individuals with high self-reported stress [10]. More recent studies, including work by Khalifa et al., have validated these findings and highlighted the need to examine population-level differences [11, 12].

A 2017 systematic review of 46 observational studies assessed associations between psychosocial factors and stroke, including only participants without prior stroke to reflect incident events [5]. However, since the publication of this review, a substantial body of new primary research has emerged, including larger cohorts and more rigorous longitudinal data. Consequently, the existing evidence base requires updating. Building on this work, the present study incorporates these recent primary studies published through 2026 to provide a more contemporary and statistically robust estimation of the association between psychosocial stress and first-ever stroke.

Accurately refining these risk estimates requires a clear understanding of the temporal relationship between psychosocial exposures and subsequent stroke. Previous reviews often included individuals with a prior history of stroke [10, 11], complicating interpretation because post-stroke sequelae—such as mood disturbances or disability—can influence self-reported stress levels, introducing the possibility of reverse causation [13]. Focusing on stroke-free individuals at baseline is therefore a sound approach to assess psychosocial stress as a potential contributing factor to first-ever incident stroke while minimizing the potential impact of reverse causation.

## Methodology

This systematic review and meta-analysis was conducted and reported according to the PRISMA 2020 guidelines [14]. The review also adhered to the MOOSE checklist for Meta-analysis of Observational Studies in Epidemiology [15].

### Search strategy

A comprehensive literature search was independently conducted by two reviewers across the following electronic databases: PubMed, Scopus, Web of Science, and Ovid. The search covered the period from database inception to January 1, 2026. The search strategy combined controlled vocabulary and free-text terms related to psychosocial stress and stroke as follows: (“psychosocial stress” OR “psychological stress” OR “mental stress” OR “work stress” OR “chronic stress” OR “job strain” OR “social stress” OR “life stress” OR “stressful life events” OR “emotional stress”) AND (“stroke” OR “cerebrovascular accident” OR “cerebral infarction” OR “ischemic stroke” OR “hemorrhagic stroke” OR “transient ischemic attack” OR “cerebral hemorrhage”). The search was restricted to studies published in the English language because translation resources were not available. In addition to electronic database searching, the reference lists of included studies and relevant prior reviews were manually screened. Forward citation tracking was also performed for key prior reviews to identify additional eligible studies.

### Eligibility criteria

This systematic review included studies that met prespecified inclusion criteria. Eligible studies employed an observational design—prospective cohort, retrospective cohort, or case–control — and assessed self-reported exposure to stressful life events (SLE), perceived psychosocial stress, work stress, or high-strain work. Studies were required to report quantitative risk estimates for stroke outcomes with 95% confidence intervals (CIs), comparing participants with higher stress exposure to those with lower or no exposure. Only studies reporting stroke as an outcome and stress as a risk factor were considered. Stroke was broadly defined to include ischemic stroke, hemorrhagic stroke, subarachnoid hemorrhage, and transient ischemic attack (TIA). Studies investigating any psychosocial risk factors across psychological, vocational, behavioral, interpersonal domains were included.

The psychological domain was defined by measures of emotional distress, depression, anxiety, perceived stress, and related affective constructs. The vocational domain included occupational stressors such as job strain, work-related stress, job dissatisfaction, and high-demand/low-control work environments. The interpersonal domain included measures of social relationship quality, including social isolation, lack of social support, and interpersonal conflict. The behavioral domain included maladaptive personality traits and coping patterns, such as hostility and anger expression.

For the purposes of this review, psychosocial stress was broadly operationally defined to include not only direct measures of perceived stress or stressful life events, but also conditions and circumstances plausibly linked to chronic psychosocial or physiological stress burden. This included psychiatric diagnoses associated with heightened sympathetic and HPA-axis activation (e.g., schizophrenia, bipolar disorder), socioeconomic and educational disadvantage as proxies for chronic social stress exposure, and occupational physical stressors such as workplace noise. This broader operational definition was adopted to comprehensively capture the multidimensional nature of psychosocial stress as it has been conceptualized in prior literature [5, 10].

Studies were excluded if they involved participants with a prior history of stroke. In addition, randomized controlled trials, reviews, editorials, case reports, animal studies, non-English publications, and duplicate or overlapping datasets were excluded to ensure consistency and avoid double-counting.

### Study selection process

All screening steps were independently performed by two reviewers, including title, abstract, and full-text screening. Any disagreements arising at any stage of the study selection process were resolved through discussion and consensus with a third reviewer.

## Data extraction

Data from all included studies were independently extracted by two reviewers using a standardized data extraction form. Discrepancies between reviewers were resolved through discussion or consultation with a third reviewer to ensure accuracy. Extracted study characteristics included the first author, publication year, country, and study design; participant details such as sample size, age, and sex; exposure information including type of stress; outcome measures, which included risk estimates with 95% CIs; and confounders included in the statistical models. Not all included studies contributed data to all four domains; each study was included only in the domain-specific analysis or analyses corresponding to the exposure(s) it assessed.

### Quality assessment

The methodological quality of each included study was independently assessed by reviewers using the Newcastle–Ottawa Scale (NOS). This tool is designed to assess the quality of non-randomized studies in meta-analyses by evaluating selection, comparability, and outcome ascertainment.

Studies were awarded points for high-quality elements within these categories. A maximum of nine points can be awarded to a study. We classified studies with scores of 7–9 as high quality, 4–6 as moderate quality, and 0–3 as low quality/high risk of bias. This assessment was performed to evaluate the risk of bias for each included study.

### Data synthesis

Meta-analysis was performed using R software version 4.4.3. Effect sizes from individual studies were extracted as Hazard Ratios (HR), Relative Risks (RR), or Odds Ratios (OR) with their corresponding 95% confidence intervals (CIs). For the primary analysis of cohort studies, Hazard Ratios were used as the common measure of association. Studies reporting Relative Risks (RR) were included in the primary analysis under the assumption that RR approximates HR in the context of stroke incidence. We pooled the effect estimates using the generic inverse variance method with a random-effects model to account for anticipated heterogeneity between studies. For case–control studies and cohort studies reporting Odds Ratios, a separate pooled analysis was conducted to calculate a pooled OR. Heterogeneity among studies was assessed using the Cochran Q test and quantified using the I2 statistic. Significant heterogeneity was defined as a *P*-value < 0.10 for the Q test or an I2 value > 50%. To test the consistency of our findings and explore sources of heterogeneity, we conducted both sensitivity and subgroup analyses. First, as a sensitivity analysis, we repeated the primary pooling after excluding studies identified as statistical outliers to observe changes in the pooled estimate and the reduction in heterogeneity. Subsequently, we performed subgroup analyses to evaluate specific variations in the data. We stratified the analysis by the type of effect measure reported (analyzing studies reporting HRs separately from those reporting RRs) to determine whether the inclusion of RRs altered the results. Additionally, we analyzed the data by gender (male vs. female) to compare the association between psychological factors and stroke risk between men and women. A test for subgroup differences was used to compare these groups. To further evaluate the robustness of the pooled effect estimate against potential small-study effects while accounting for substantial between-study heterogeneity, a limit meta-analysis was performed. A two-sided P-value < 0.05 was considered statistically significant throughout.

## Results

### Study selection and characteristics

**Fig. 1:**
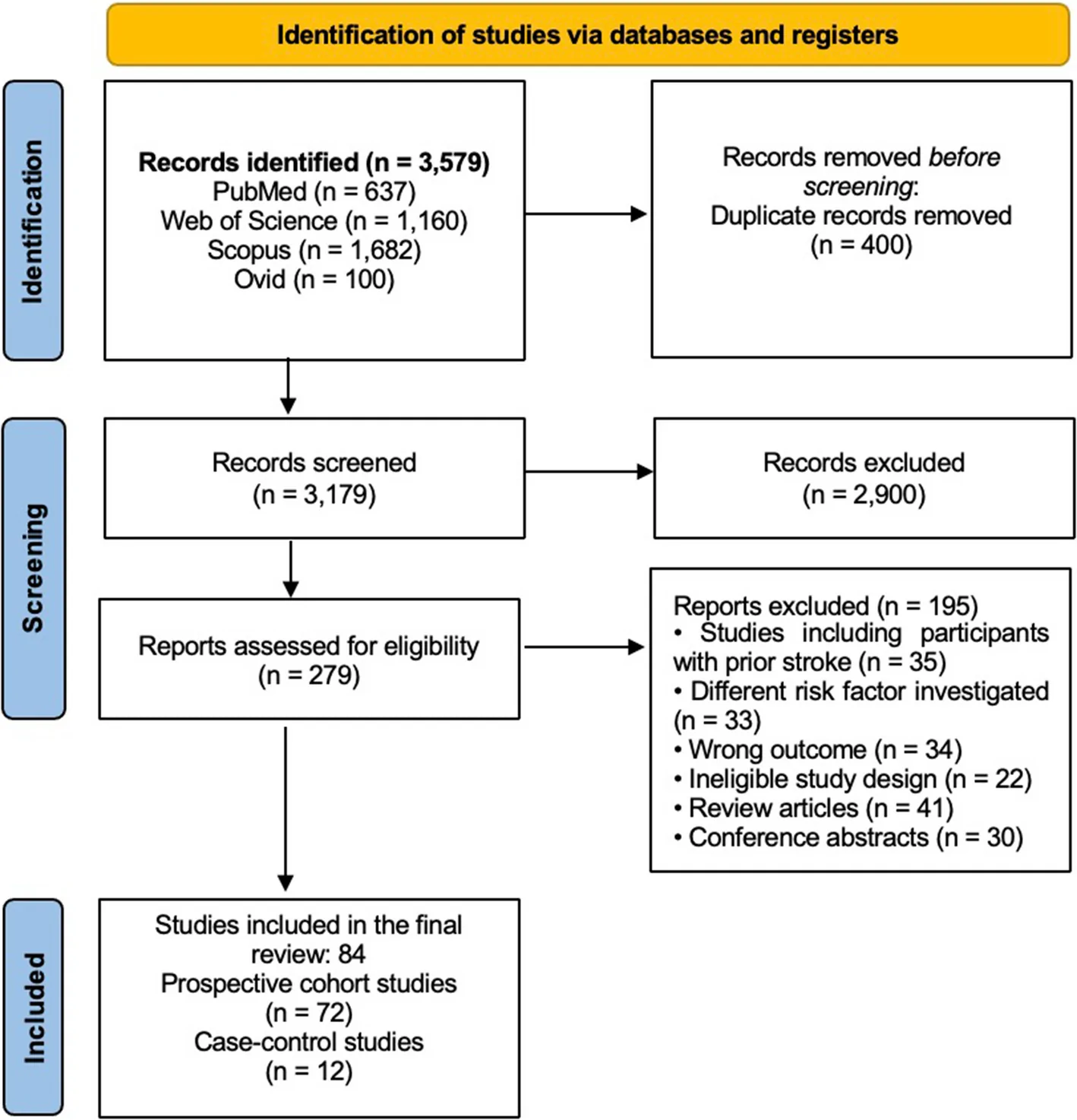
PRISMA Flow Diagram

Following the initial screening of titles and abstracts, 3,179 unique records were identified. A total of 279 full-text articles were assessed for eligibility. Ultimately, 84 studies, comprising 72 prospective cohort studies [12, 16–86] and 12 case–control studies [9, 13, 87–96], met the inclusion criteria and were included in the systematic review, encompassing a total of 4,128,167 participants (Fig. 1). The included studies investigated four categories of psychosocial factors: psychological, interpersonal, behavioral, and vocational. A summary of the characteristics and findings of the included studies is presented in Table 1. Depression was the most frequently investigated psychological factor, as summarized in Table 2. The baseline characteristics of the case–control and cohort studies are presented in Appendix Tables T.1 and T.2, respectively.

**Table 1:** Summary table of included studies

Study ID	Country	Study design	Type of exposure	Conclusion
Abel 1999	USA	Case Control	Psychological	No significant association between ischemic stroke and social readjustment stress
Almas 2015	Sweden	Prospective Cohort	Psychological	Moderate or severe depression is related to an increased risk of CVD
Blomstrand 2022	Sweden	Prospective Cohort	Psychological, educational, socioeconomic	Low education was associated with stroke
Cummings 2016	USA	Prospective Cohort	Socioeconomic, behavioural, educational	Comorbid stress and depression increased cardiovascular risk in diabetes
Fenger-Grøn 2020	Denmark	Prospective Cohort	Psychological, educational, socioeconomic status	Partner bereavement modestly increased ischemic and hemorrhagic stroke risk
Henderson 2013	USA	Prospective Cohort	Psychological stress	Higher psychosocial distress was significantly associated with increased risk of incident stroke
Kuper 2007	Sweden	Prospective Cohort	Educational, interpersonal	Lower education increased stroke risk, largely explained by conventional risk factors
Araki 2004	USA	Prospective Cohort	Psychological	Low well-being predicted stroke risk in elderly patients with diabetes
Arbelaez 2007	USA	Prospective Cohort	Psychological	Depressive symptoms independently increased incident stroke risk
Bos 2008	Netherlands	Prospective Cohort	Psychological	The presence of depressive symptoms is a strong risk factor for stroke in men but not in women
Curkendall 2004	Canada	Retrospective Cohort	Psychological	Schizophrenia was associated with increased cardiovascular morbidity and mortality
Eurelings 2013	Netherlands	Prospective Cohort	Psychological	Apathy, not depression, independently predicted cardiovascular disease risk
Öhlin 2004	Sweden	Prospective Cohort	Psychological	Chronic stress independently increased cardiovascular disease and fatal stroke risk, mainly in men
Surtees 2007	UK	Prospective Cohort	Social and psychological	Stress adaptive capacity is a potentially important candidate risk factor for stroke
Bergh 2014	Sweden	Prospective Cohort	Psychological	Low stress resilience was associated with increased stroke risk
Blomstrand 2014	Sweden	Prospective Cohort	Psychological, behavioral, socioeconomic	Low education independently increased ischemic stroke risk
Egido 2012	Spain	Case Control	Psychosocial, behavioral	Psychophysical stress independently increased stroke risk; no sex differences were observed
Everson-Rose 2014	USA	Prospective Cohort	Psychological	Stress, hostility, and depression independently increased stroke or TIA risk
Feller 2013	Germany	Prospective Cohort	Psychological	Lower life satisfaction was associated with increased risk of stroke and myocardial infarction during follow-up
Hamano 2014	Sweden	Prospective Cohort	Psychological	Depression was associated with increased odds of stroke
Lahti 2012	Finland	Prospective Cohort	Psychological	Stroke hospitalization and mortality were not significantly increased overall, though women showed marginally increased stroke mortality
Lee 2008	Taiwan	Prospective Cohort	Psychological	Over 5 years, depressed patients had a > fivefold higher hazard of stroke than matched controls
Lin 2007	Taiwan	Prospective Cohort	Psychiatric	Strokes occurred more in bipolar patients than controls
Gafarov 2013	Russia	Prospective Cohort	Psychosocial	Low social support increased myocardial infarction and stroke risk in women
Gafarov 2013	Russia	Prospective Cohort	Psychological	Depression markedly increased myocardial infarction and stroke risk in women
Jood 2017	Sweden	Case Control	Occupational	Adverse psychosocial work conditions were associated with increased stroke risk
Kivimaki 2007	Sweden	Prospective Cohort	Occupational	Job strain increased cardiovascular risk, particularly in younger employees
Kornerup 2010	Denmark	Prospective Cohort	Psychological	Vital exhaustion increased ischemic stroke risk in women only
Nielsen 2005	Denmark	Prospective Cohort	Psychological	High psychological stress was associated with increased stroke risk
Reddin 2022	32 countries	Case Control	Psychosocial	Suggest an association between psychosocial stress and stressful life events and an increased risk of all stroke. This association is consistent for ischemic and hemorrhagic stroke types
Riahi 2023	USA	Prospective Cohort	Psychosocial, psychological	Higher levels of chronic stress are associated with greater risk of incident cardiovascular disease
Santosa 2021	21 countries	Prospective Cohort	Psychosocial	Higher psychosocial stress increased cardiovascular disease and stroke risk
Majed 2012	France, Northern Ireland	Prospective Cohort	Psychological	Depressive symptoms increased long-term stroke risk, especially ischemic stroke
Mejia-Lancheros 2014	Spain	Prospective Cohort	Psychosocial factors	Adults with low educational level had a higher risk of stroke. Depression and low social support were not associated with CVD incidence
Nabi 2010	Finland	Prospective Cohort	Psychological	In this population of adult men and women, low level of pessimism had a robust association with reduced incidence of stroke
Nilsson 2004	Denmark	Prospective Cohort	Psychological	Severe depression was associated with increased cerebrovascular disease risk
Salaycik 2006	USA	Prospective Cohort	Psychological	Depressive symptoms independently increased incident stroke or TIA risk
Shirai 2009	Japan	Prospective Cohort	Psychological	Low life enjoyment increased cardiovascular disease risk and mortality
Schiöler 2015	Sweden	Prospective Cohort	Psychosocial	No significant associations between psychosocial work environment and ischemic stroke, and the associations between job demands and control and CHD were inconsistent and weak
Sumner 2015	USA	Prospective Cohort	Psychological, physical	Trauma exposure and elevated PTSD symptoms may increase risk of CVD, including stroke, in this population of women
Tezuka 2019	Japan	Prospective Cohort	Psychological	Anger expression increased cardiovascular disease risk among urban residents
Williams 2002	USA	Prospective Cohort	Psychological	Trait anger was associated with an increased risk for incident stroke in the ARIC study among younger participants and those with higher HDL cholesterol levels
Xu 2022	Denmark, Finland, Sweden	Prospective Cohort	Psychological, occupational	Individuals with high levels of workplace psychosocial resources across horizontal and vertical dimensions have a lower risk of CVD, particularly cerebrovascular disease
André-Petersson 2007	Sweden	Prospective Cohort	Occupational, interpersonal	For women, low levels of social support at work were associated with an increased risk of an MI and stroke. No associations were found among men
Behymer 2025	USA	Case Control	Psychological	Higher levels of all stress subtypes were associated with increased odds of ICH
Everson 2001	Finland	Prospective Cohort	Interpersonal	Excessive sympathetic reactivity to stress may be etiologically important in stroke, especially ischemic strokes, and low socioeconomic status confers added risk
Smoller 2007	USA	Prospective Cohort	Psychosocial	Panic attacks independently increased cardiovascular disease risk in postmenopausal women
Kuan-Yi Tsai 2012	Taiwan	Prospective Cohort	Psychosocial	that patients with schizophrenia were at a significantly higher risk for stroke and post-stroke all-cause mortality
Yan 2012	USA	Prospective Cohort	Psychological	Living in a socioeconomically disadvantaged neighborhood is associated with an increased risk of ischemic stroke in white older adults
Gilum 2003	USA	Prospective Cohort	Socioeconomic	Risk for stroke and post-stroke all-cause mortality
Honjo 2015	Japan	Prospective Cohort	Socioeconomic	Multiple social roles reduced stroke risk in highly educated working women
Fujino 2007	Japan	Prospective Cohort	Occupational	Occupational noise increased intracerebral hemorrhage mortality risk in hypertensive workers
Iso 2002	Japan	Prospective Cohort	Psychological	Perceived mental stress increased stroke mortality in women
Jackson 2018	Australia	Prospective Cohort	Psychological	Psychological distress dose-dependently increased myocardial infarction and stroke risk
May 2002	Wales	Prospective Cohort	Psychological	Psychological distress is a predictor of fatal ischemic stroke but not of nonfatal ischemic stroke or TIA
O’Donnell 2010	22 countries	Case Control	Interpersonal, Occupational	Psychosocial stress was a major modifiable risk factor for stroke
Sarfo 2022	Ghana and Nigeria	Case Control	Interpersonal, occupational	Psychosocial stress and cardiac disease were associated with stroke occurrence among young West Africans
Sarfo 2018	Ghana and Nigeria	Case Control	Interpersonal, occupational	Psychosocial stress and cardiac disease were associated with stroke occurrence among young West Africans
Surtees 2008	UK	Prospective Cohort	Psychological	Higher psychological distress (lower MHI-5) was associated with increased incident stroke
Truelsen 2003	Denmark	Prospective Cohort	Psychological	Self-reported high stress was associated with a higher risk of fatal stroke
Zaitsu 2019	Japan	Case Control	Occupational	In Japanese society, managers and professionals may experience a higher risk for CHD
Lawrence 1996	USA	Retrospective Cohort	Psychological	Extreme psychological and physical stress during captivity may have long-term vascular consequences
Honjo 2008	Japan	Prospective Cohort	Educational, Socioeconomic	A potential benefit of multiple social roles was suggested for stroke risk reduction among highly educated working women
Li 2008	Sweden	Prospective Cohort	Socioeconomic	Incidence of stroke, stroke recurrence, and case-fatality increased with decreasing socioeconomic status;
McFadden 2009	UK	Prospective Cohort	Socioeconomic	An inverse relationship was observed between social class and stroke incidence
Veronesi 2010	Italy	Prospective Cohort	Educational	The association between education and cardiovascular risk seems to vary by gender
Ohira 2009	Japan	Prospective Cohort	psychological	Higher perceived psychological stress was associated with an increased risk of total stroke
Eriksson 2018	Sweden	Prospective Cohort	Occupational	Exposure to occupational noise was associated with an increased risk for CHD. None of the analyzed variables were related to increased risk for stroke
Uchiyama 2005	Japan	Prospective Cohort	Occupational	Active jobs and high-strain jobs were associated with increased risk of CVE for treated hypertensive workers
Tsutsumi 2009	Japan	Prospective Cohort	Occupational	Occupational stress related to job strain was associated with incidence of stroke among japanese men
Harmsen 2006	Sweden	Prospective Cohort	Psychological, Interpersonal, occupational	Psychological stress is a significant predictor of stroke. Socioeconomic status did not emerge as a statistically significant predictor of stroke
Gallo 2014	USA	Prospective Cohort	Psychological	Stress burden was significantly associated with increased risk of stroke and TIA
Glover 2020	USA	Prospective Cohort	Psychological	Higher GSS may be a risk factor for developing CHD among women; however, it appears to be protective of stroke among women
Harmsen 1990	Sweden	Prospective Cohort	Psychological	Independent risk factors for non-hemorrhagic stroke were high diastolic blood pressure, smoking, severe psychological stress
Metcalfe 2005	Scotland	Prospective Cohort	Psychological, socioeconomic	Stroke risk was particularly linked to childhood socioeconomic disadvantage
Ikeda 2008	Japan	Prospective Cohort	Interpersonal, Social	Low social support increased stroke mortality risk in men
Nagayoshi 2014	USA	Prospective Cohort	Interpersonal, Social	Having a small social network was associated with an excess risk of incident stroke
Riaz 2016	Bangladesh	Case Control	Psychological	Psychosocial stress was a risk for both ischemic and hemorrhagic stroke
Engström 2004	Sweden	Prospective Cohort	Interpersonal, Social	The incidence of stroke is increased in divorced and widowed individuals
Ohira 2001	Japan	Prospective Cohort	Psychological	higher level of depressive symptoms had higher risks of CHD and ischemic stroke
Khan 2023	32 countries	Case Control	Psychological	Psychosocial stress is a risk factor for stroke in those younger than 45 years of age
Jood 2009	Sweden	Case Control	Psychological	Permanent stress ≥ year increases ischemic stroke risk
Prasad 2020	India	Case Control	Psychological	Acute psychological stress is an independent triggering factor for stroke
Eng 2003	USA	Prospective Cohort	Psychological	Outward expression of anger appeared protective against stroke

**Table 2:** Frequency of Psychological Factors

Frequency	Suggested Stressor
17	Depression
8	Chronic Stress
6	Psychological Distress
5	Mental Stress
3	Neighborhood Socioeconomic Condition
3	Schizophrenia
2	Life Satisfaction
2	Perceived level of Life Enjoyment
2	Psychological Stress
2	Race
2	Self-Reported Stress
2	Others
1	Anger Expression
1	Bipolar
1	Former Prisoners of war
1	Panic Attack
1	Severe Psychological Stress
1	Social Readjustment
1	Stress Resilience
1	Well-Being
1	Anxiety
1	Level of Pessimism
1	Self-Perceptiveness
1	Sense of Coherence
1	Social Class

### Risk of bias assessment

The methodological quality of the included observational studies of both case–control and cohort designs was evaluated using the NOS. Overall, the studies demonstrated high quality, with total NOS scores ranging from 7 to 9 out of 9. Most studies performed well in the selection and exposure/outcome domains, and the majority achieved adequate comparability. No study was excluded based on quality assessment, and the overall risk of bias across all included studies was considered low.

Appendix Tables T.3 and T.4 summarize the NOS assessment for case control, and cohort studies respectively.

### Meta-analysis findings

Across all four psychosocial domains examined — psychological, interpersonal, behavioral, and vocational — pooled estimates consistently pointed toward an increased risk of stroke, with cohort-study findings reaching statistical significance in all four categories. The strength and consistency of this association varied by domain, however. Psychological and vocational factors showed the largest evidence base, supported by a substantial number of cohort studies (48 and 20, respectively) with significantly increased risk across both hazard-ratio and odds-ratio designs. Interpersonal and behavioral factors were represented by fewer studies (7 and 3 cohort studies, respectively); their cohort-based estimates reached significance. The corresponding interpersonal case–control subset did not reach significance, whereas the behavioral case-control subset showed a significant association. Heterogeneity was substantial across most domains (I^2^ ranging from 46 to 95%), with the exception of behavioral factors, where heterogeneity was low (I^2^ = 0–13%) but based on few studies. These domain-specific findings are presented in detail below.

### Psychological factors (P Factors)

#### Cohort studies

**Fig. 2:**
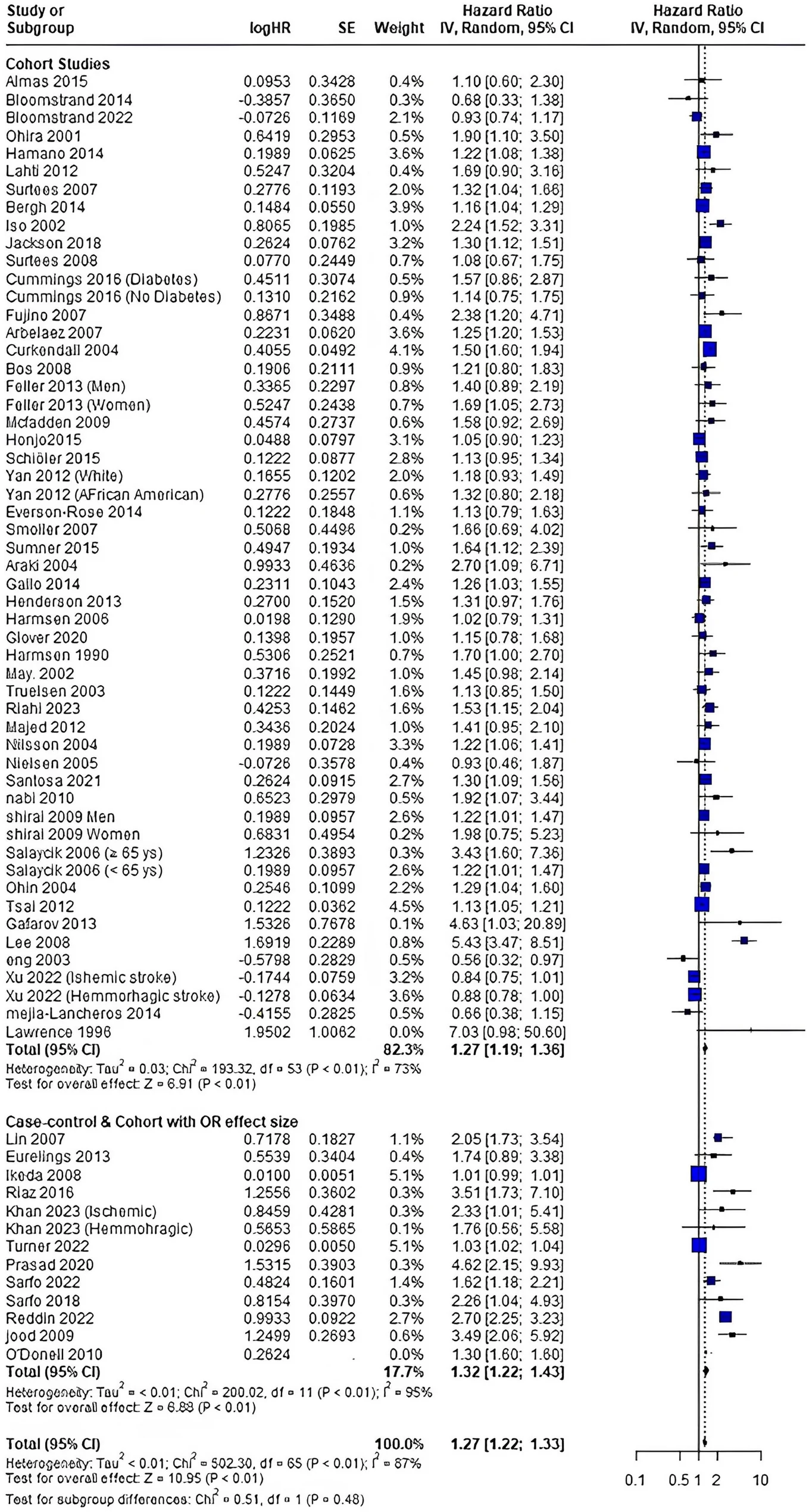
Psychological Risk Factors Forest Plot

A total of 48 studies demonstrated an increased risk of stroke among patients with psychological risk factors (HR = 1.27, 95% CI [1.19:1.36], *P* < 0.01); with significant heterogeneity (I^2^ = 73%, *P* < 0.01), as shown in Fig. 2**.** This heterogeneity was notably reduced through sensitivity analysis after excluding Lawrence (1996) [76], Xu 2022 [86], Gafarov (2013) [81], Lee (2008) [68], and Eng (2003) [19], while maintaining a significantly increased risk of stroke (HR = 1.26, 95% CI [1.20:1.33], *P* < 0.01); (I^2^ = 45.8%, *P* < 0.01) Appendix.Fig. 1.

#### Case control, and cohort studies reporting OR

A significant increase of the risk of stroke was also noticed among patients with psychological factors (OR = 1.32, 95% CI [1.22:1.43], *P* < 0.01); (I^2^ = 73%, *P* < 0.01) as shown in Fig. 2.

#### Publication bias assessment

Funnel plot inspection suggested asymmetry, which was confirmed by Egger’s test (*P* < 0.0001), indicating publication bias (Appendix A.3). Limit meta-analysis, applied to account for small-study effects and heterogeneity, yielded an adjusted pooled hazard ratio of 1.14 (95% CI: 1.04–1.25, *P* < 0.01), which was attenuated compared to the unadjusted estimate of 1.27 (95% CI: 1.19–1.36, *P* < 0.01), though the association remained statistically significant. The test for small-study effects was significant (*P* < 0.01), while substantial residual heterogeneity persisted beyond this adjustment (*P* < 0.01), suggesting that heterogeneity, rather than small-study effects alone, remained the dominant source of variability across studies.

#### Vocational factors (V Factors)

**Fig. 3:**
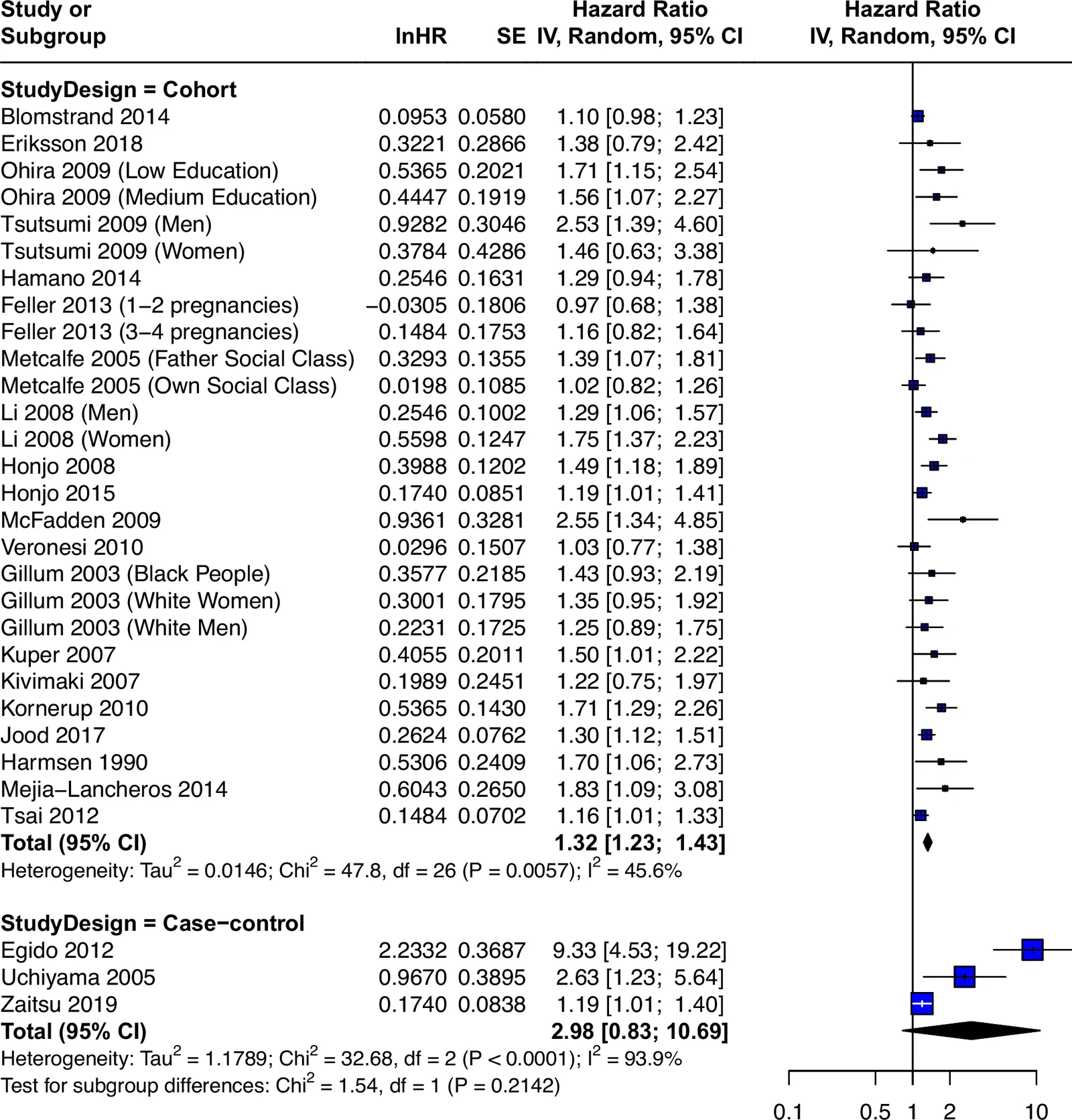
Vocational Risk Factors Forest Plot

A significant increase in stroke risk was noticed in subjects exposed to vocational risk factors among 20 cohort studies (HR = 1.32, 95% CI [1.23:1.43], *P* < 0.01); (I^2^ = 46%, *P* < 0.01), however, 3 case control studies did not report significant association (OR = 2.98, 95% CI [0.83:10.69], *P* = 0.09); (I^2^ = 94%, *P* < 0.01) as shown in Fig. 3**.**

#### Publication bias assessment

Visual inspection of funnel plot (Appendix Fig. 6) indicated asymmetry, which was confirmed by Egger’s test (*P* < 0.01). Limit meta-analysis for the vocational domain yielded an adjusted pooled hazard ratio of 1.16 (95% CI: 1.02–1.31, *P* = 0.021), attenuated relative to the unadjusted estimate of 1.32 (95% CI: 1.23–1.43, *P* < 0.01), though the association remained statistically significant after adjustment. The test for small-study effects was significant (*P* < 0.01), whereas residual heterogeneity beyond this adjustment was no longer significant (*P* = 0.15), suggesting that small-study effects may largely account for the heterogeneity observed in this subgroup.

#### Interpersonal factors (I Factors)

**Fig. 4:**
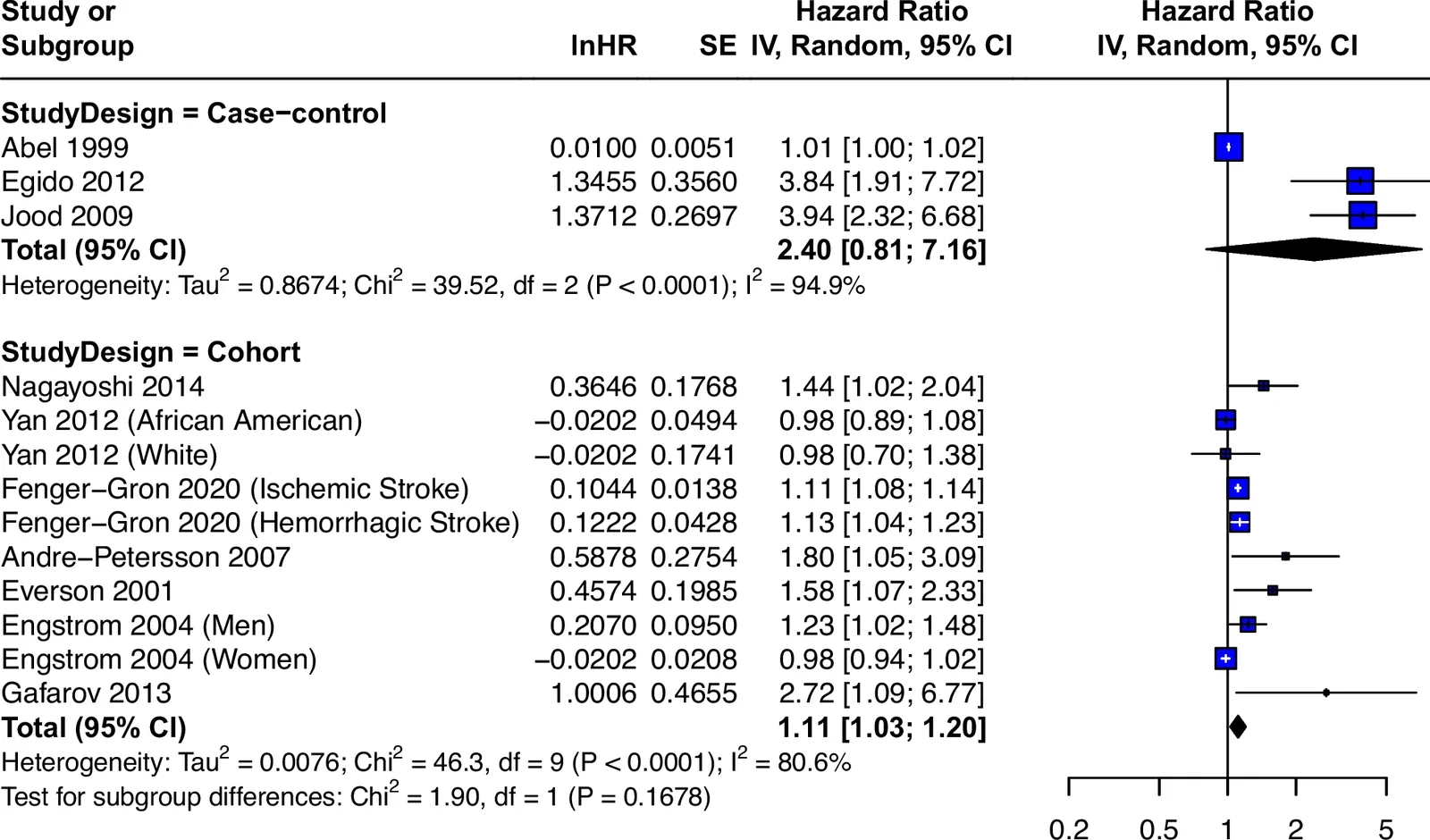
Interpersonal Risk Factors Forest Plot

A total of 7 cohort studies demonstrated a significant association between the interpersonal factors and stroke risk (HR = 1.11, 95% CI [1.03:1.2], *P* < 0.01); with significant heterogeneity (I^2^ = 81%, *P* < 0.01). However, 3 case control studies did not show significant association between stroke risk and interpersonal risk factors (OR = 2.4, 95% CI [0.81:7.16], *P* = 0.12); (I^2^ = 95%, *P* < 0.01) as shown in Fig. 4.

#### Behavioral factors (B Factors)

The analysis indicated a significant association between behavioral risk factors and stroke incidence in 3 cohort studies (HR = 1.51, 95% CI [1.05:2.15], *P* = 0.02); (I^2^ = 13%, *P* = 0.33), and case control studies (OR = 2.37, 95% CI [1.4:4], *P* < 0.01); (I^2^ = 0%, *P* = 0.66) as shown in Appendix.Fig. 4.

## Subgroup analyses within psychological factors

### Effect measure

**S**ubgroup analysis based on the effect measure type (HR, OR, and RR) demonstrated a consistently increased risk of stroke among patients with psychological risk factors. Studies reporting HR demonstrated a significant association (HR = 1.27, 95% CI [1.18:1.37], *P* < 0.01); (I^2^ = 74%, *P* < 0.01). Similarly, studies using OR showed a significant increase in stroke risk (OR = 1.31, 95% CI [1.22:1.41], *P* < 0.01); (I^2^ = 94%, *P* < 0.01), however, studies reporting RR did not show significant trend towards increased stroke risk (RR = 1.43, 95% CI [0.98:2.11], *P *= 0.07); (I^2^ = 42%, *P* = 0.16), based on only four studies contributing a small proportion of the overall weight (2.9%), as shown in Appendix Fig. 2. Despite this difference in statistical significance across effect-measure subgroups, the formal test for subgroup differences was non-significant (*P* = 0.74), indicating that the lack of significance in the RR subgroup was more likely attributable to its small number of studies (k = 4) and correspondingly wide confidence interval, rather than a true difference in effect related to study methodology or effect-measure type.

### Gender

Subgroup analysis by gender revealed a significant increase in stroke risk among males with psychological risk factors (HR = 1.19, 95% CI [1.07:1.32], *P* < 0.01); (I^2^ = 0%, *P* = 0.66). In contrast, no significant association was observed among females (HR = 0.86, 95% CI [0.58:1.28], *P* = 0.46); (I^2^ = 68%, *P* = 0.01). The test for subgroup differences was not statistically significant (*P* = 0.13), indicating that the association between psychological risk factors and stroke risk did not significantly differ between males and females as shown in Appendix.Fig. 3.

## Discussion

### Overview of findings

Our meta-analysis included data from 84 studies, comprising 72 prospective cohort studies and 12 case–control studies, collectively encompassing 4,128,167 participants. This represents the most comprehensive synthesis to date to quantify the influence of psychosocial factors on stroke risk.

The pooled data from our meta-analyses indicate a significant association between Psychosocial stress and stroke risk. To facilitate a structured synthesis, psychosocial exposures were categorized into four main domains: psychological, vocational, interpersonal, and behavioral. The psychological domain focused on distress-related manifestations, anxiety-related traits, perceived stress, and general emotional distress. Vocational exposures encompassed occupational strain, work-related stress, and job dissatisfaction. Interpersonal exposures referred to adverse social relationships, such as social isolation, conflict, or lack of support, while behavioral exposures included maladaptive personality traits or coping patterns, such as hostility and anger expression.

### Psychological factors

Our analysis of 48 cohort studies demonstrated that individuals with psychological risk factors—including depression, anxiety, perceived stress, and emotional distress—had a 27% higher risk of first-ever stroke compared to those without these exposures. These results align with prior evidence, such as Lightbody et al. [5] (39% increased risk), Khalifa et al. [11] (46% increased risk), and Truelsen et al. [67] (nearly twofold higher risk of fatal stroke among highly stressed individuals), supporting the consistency of this association across diverse populations. The slightly lower effect estimate in our analysis may be attributed to the inclusion of a larger number of studies that specifically excluded participants with a prior history of stroke, thereby reflecting incident events more accurately. Sensitivity analyses excluding studies that contributed to significant heterogeneity (Lawrence (1996) [76], Xu 2022 [86], Gafarov (2013) [81], Lee (2008) [68], and Eng (2003) [19]) confirmed the robustness of these findings.

Chronic psychological stress may increase stroke risk through biological pathways, including activation of the hypothalamic–pituitary–adrenal (HPA) axis and sympathetic nervous system, resulting in sustained elevations of cortisol and catecholamines, which promote hypertension, endothelial dysfunction, atherosclerosis, and prothrombotic states [97, 98]. Stress also contributes to systemic inflammation, reflected in elevated C-reactive protein and interleukin-6, further exacerbating vascular injury [99]. These mechanisms highlight the potential value of integrating psychological well-being into stroke prevention strategies.

### Other psychosocial factors

Our analysis of 20 cohort studies demonstrated that exposure to occupational stressors—including work strain, job dissatisfaction, and high demand—was associated with a 32% higher risk of first-ever stroke during follow-up. These findings are broadly consistent with previous studies, such as Lightbody et al. [5] (35% increased risk) and Fransson et al. [100] (24% higher risk of ischemic stroke after adjustment for age and sex).

Our analysis of seven cohort studies indicated that adverse interpersonal factors, such as social isolation or conflict, were associated with an 11% increased risk of first-ever stroke. These results align with previous research, including Lightbody et al. [5] (16% increased risk) and Ikeda et al. [72], which reported that low social support increased stroke mortality in men. Differences in effect sizes may reflect our focus on stroke-free participants at baseline. However, social support was not significantly associated with stroke incidence, suggesting that social support may influence stroke outcomes and prognosis more than the initial occurrence of stroke.

Although only three cohort studies examined behavioral risk factors, our meta-analysis showed that individuals exhibiting maladaptive behaviors—such as hostility, anger expression, or poor coping—had a 51% higher risk of first-ever stroke. This finding should be interpreted cautiously because it was derived from a small evidence base and is therefore preliminary compared with the estimates for psychological and vocational factors. Behavioral patterns may constitute modifiable risk factors warranting further research.

Psychosocial factors also influence stroke risk indirectly through behavioral pathways, as high stress is associated with unhealthy habits including smoking, excessive alcohol consumption, physical inactivity, and poor diet, each independently elevating stroke risk [10].

Comparing across the four psychosocial domains, behavioral traits demonstrated the largest pooled effect estimate (HR = 1.51), followed by vocational stressors (HR = 1.32), psychological factors (HR = 1.27), and interpersonal stressors (HR = 1.11). This gradient may partly reflect differences in underlying physiological mechanisms. Hostility and anger expression have been linked to prolonged cardiovascular reactivity, including longer-lasting blood pressure responses during and after anger-provoking stimuli [101]. Alternatively, this gradient may partly reflect differences in statistical precision and study count; the behavioral domain comprised only three studies, and small-study effects may inflate pooled estimates in domains with limited evidence. The comparatively modest and more heterogeneous effect observed for interpersonal factors may also reflect the difficulty of consistently operationalizing constructs such as social isolation across diverse study contexts, compared with more standardized instruments used to assess depression or occupational strain.

### Strengths

Our meta-analysis has several key strengths that enhance the clarity, reliability, and applicability of its findings. First, it incorporates data from 84 studies encompassing diverse populations and settings, providing substantial statistical power and broad generalizability. Second, by excluding participants with a prior history of stroke, the analysis focuses exclusively on first-ever stroke events, thereby strengthening causal inference and minimizing the risk of reverse causation.

Third, psychosocial factors were examined across four well-defined domains—psychological, vocational, interpersonal, and behavioral—allowing for a comprehensive assessment of psychosocial risk and facilitating targeted prevention strategies. Finally, results remained consistent across multiple sensitivity analyses, even after removing potential outliers or studies with high heterogeneity, underscoring the stability of the observed associations.

### Limitations

Despite its strengths, our meta-analysis has several limitations that should be considered. Substantial heterogeneity was observed across the included studies, driven by differences in study design, definitions and measurement of psychosocial factors, follow-up duration, and the extent of adjustment for confounders, which reduces the precision of pooled estimates and necessitates caution when generalizing the findings. Therefore, the pooled estimates should not be interpreted as a single universal effect applicable to all populations. Rather, they represent average associations across studies that differed in population characteristics, psychosocial exposure definitions, follow-up duration, stroke ascertainment, and covariate adjustment. This heterogeneity limits the precision and clinical transportability of any single pooled estimate.

Additionally, our meta-analysis combined studies reporting RR and HR for the outcome of psychological factors under the assumption that these measures approximate one another for stroke outcomes. While subgroup analysis showed that RR-only studies did not reach statistical significance (RR = 1.43, 95% CI 0.98–2.11, *P* = 0.07), this subgroup comprised only four studies with limited statistical power, and the formal test for subgroup differences was non-significant (*P* = 0.74), suggesting the pooled effect estimate is not meaningfully driven by differences in effect-measure type. Nonetheless, caution is required given the small number of RR-based studies available. Residual confounding remains a concern, as important variables such as socioeconomic status, comorbidities, medication use, and genetic predisposition were not consistently accounted for, potentially influencing the observed associations. Moreover, clinically relevant variables such as age, comorbidities, and stroke severity were not consistently defined or reported across studies, precluding meaningful quantitative moderator analyses to explore their potential influence on the pooled associations. Evidence of publication bias, detected through funnel plot asymmetry and Egger’s test, suggests that studies with statistically significant results may be overrepresented, possibly leading to an overestimation of effect sizes.

Additionally, the heavy reliance on self-reported psychosocial measures introduces the potential for recall bias, social desirability bias, and general measurement error. Furthermore, methodological details such as the timing of exposure assessment and the specific instruments used to measure psychosocial factors were inconsistently reported across the included studies, limiting our ability to comprehensively extract and synthesize this information. Some studies assessed exposures only at baseline, limiting the ability to account for changes in stress levels or life circumstances over time. Generalizability is further constrained because the majority of studies were conducted in high-income countries, where psychosocial stressors, healthcare access, and stroke epidemiology differ from low- and middle-income settings. The English-language restriction may have introduced language bias and may have led to omission of relevant non-English studies.

### Future research directions

Future research should address several critical areas to deepen understanding and inform effective stroke prevention strategies. Mechanistic studies are needed to elucidate the biological pathways linking psychosocial stress to stroke, including neuroendocrine dysregulation, inflammatory cascades, autonomic imbalance, and potential epigenetic modifications, which could guide the development of targeted interventions. Longitudinal studies with repeated measurements of psychosocial exposures are essential to capture the dynamic and cumulative nature of stress over time. Additionally, the use of standardized and validated instruments, such as the Perceived Stress Scale or the Job Strain Index, would improve comparability and reproducibility across studies.

Further research should explore how demographic and socioeconomic factors—such as age, sex, ethnicity, and socioeconomic status—modify the relationship between psychosocial stress and stroke, as early evidence suggests potential differences, particularly between men and women. Intervention studies represent a crucial next step; evaluating the impact of stress-reduction programs, workplace modifications, and psychosocial support on stroke incidence would provide actionable insights for both clinicians and policymakers. Randomized controlled trials of cognitive-behavioral therapy, mindfulness-based approaches, and organizational-level workplace interventions would be particularly informative.

Expanding research to include populations from low- and middle-income countries is equally important, given differences in psychosocial stressors, healthcare infrastructure, and stroke epidemiology. Broader international collaboration will enhance the global applicability of findings and ensure representation of diverse lived experiences. Finally, integrating psychosocial measures into electronic health records and established cardiovascular risk prediction models, such as the Framingham Risk Score or QRISK, could improve risk stratification and facilitate preventive care, offering a more comprehensive approach to reducing stroke burden.

## Conclusion

Our meta-analysis provides robust evidence that psychosocial factors are independently associated with an increased risk of stroke. Psychological and vocational stressors emerged as the most consistent predictors, while behavioral and interpersonal factors demonstrated smaller but still significant effects. These findings underscore the importance of addressing mental health and workplace stress as integral components of comprehensive stroke prevention strategies.

The modifiable nature of psychosocial risk factors presents an opportunity for intervention at the individual, clinical, and population levels. Routine screening for psychosocial stressors, the integration of mental health services into cardiovascular care, and implementation of evidence-based psychosocial interventions may reduce stroke incidence and improve overall health outcomes. By highlighting the significance of psychosocial exposures as risk factors for stroke, this study emphasizes the need for continued research and clinical initiatives targeting these factors, ultimately contributing to more effective and holistic approaches to stroke prevention.

## Supplementary Information

Supplementary Material 1: Appendix T.1. Baseline Characteristic table of case-control included studies. Appendix T.2. Baseline Characteristic table of cohort included studies. Appendix T.3. Summary table of ROB assessment using the Newcastle-Ottawa Quality Assessment Scale for Case-Control studies. Appendix T.4. Summary table of ROB assessment using the Newcastle-Ottawa Quality Assessment Scale for Cohort studies. Appendix Fig.1. Psychological Factors Forest Plot after removing outliers. Appendix Fig.2. Subgroup Analysis forest Plot by Effect Measure Used. Appendix Fig.3. Gender Subgroup Analysis Forest Plot. Appendix Fig.4. Behavioral Risk Factors Forest Plot. Appendix. Fig.5 Psychological Risk Factors Funnel Plot. Appendix Fig.6. Vocational Risk Factors Funnel Plot.

## Data Availability

No new primary data were generated. All data analyzed in this study were obtained from previously published articles.
