# Supplementary material for: Psychosocial stress and risk of first-ever stroke: a systematic review and meta-analysis
Source: BMC Neurol. 2026 Sep 23;26:606. doi: 10.1186/s12883-026-05307-4 (PMC13602507; doi:10.1186/s12883-026-05307-4)
Supplement: Supplementary file 1 [file 12883_2026_5307_MOESM1_ESM.docx]

**Appendix T.1. Baseline Characteristic table of case-control included studies.**

| **Study** | **Number of participants cases:controls male** | **Age (y)** | **Cases:controls with risk factor** | **Risk factor exposure** | **Stroke outcomes** | **Number of cofounders controlled for in adjusted model** |
| --- | --- | --- | --- | --- | --- | --- |
| Egido 2012 | 150:300 | 53.8 ± 9.3* | 16:9 | Life events (Holmes & Rahe Questionnaire) | IS, HS | 8 |
| Jood 2017 | 198:396 | 54.5* | NR | Job-control-demand model, the effort-reward imbalance ERI score, and exposure to conflict at work | IS, HS | 13 |
| Reddin 2022 | 13462:13488 | 61.8 * | 2745: 1933 | Psychological stress, stressful life event measured using a standardized questionnaire of self-reported stress at home and work. | NS | 14 |
| Abel 1999 | 655:1087 | 69.8* CASES | NR | Social readjustment | IS | 4 |
| Behymer 2025 | 2964/2964 | 61.85* | NR | Financial stress, Health stress, Emotional well-being stress, Family stress | HS |  |
| O’Donnell 2010 | 3000:3000 | 63*CASES | 1:1 | Questionnaire | IS, HS | 11 |
| Sarfo 2022 | 2431:2431 | 61.1* | 1:1 | INTERSTROKE questionnaire for Psychosocial stress and depression | NS | 3 |
| Sarfo 2018 | 2118:2118 | 58.5* | 1:1 | INTERSTROKE questionnaire for Psychosocial stress and depression | NS | 37 |
| Zaitsu 2019 | 128615:999976 | 60 ± 13* | 1:7.7 | Occupational Class | NS | 2 |
| Jood 2009 | 566:593 | 56.3* | 162/46 | Single-item questionnaire for self-perceived psychological stress | IS | 9 |
| Riaz 2016 | 175:171 | NE | NR | Questionnaire on psychosocial stress, diet, smoking, etc. | IS, HS | 8 |
| Khan 2023 | 1582:1582 | 38.52* | NR | Psychosocial stress, hypertension, smoking, alcohol, diet, physical activity, obesity, dyslipidaemia, cardiac causes | IS, HS | 10 |

*mean

IS: ischemic stroke, HS: hemorrhagic stroke, NS: non-specified stroke

**Appendix T.2. Baseline Characteristic table of cohort included studies.**

| **Study Id** | **Number of participants male(%)** | **Age at baseline (y)** | **Risk Factor exposure** | **Duration of follow-up (y)** | **Number of stroke events (total)** | **Stroke outcomes** | **Number of confounders controlled for in adjusted model** |
| --- | --- | --- | --- | --- | --- | --- | --- |
| Almas 2015 | 4620 (44.67) | 41.3 | Depression and Anxious Distress | 10 | 228 | IS, HS | 16 |
| Bloostrand 2022 | 146 (10) | 60-82 | Mental Stress | 44 | 337 | IS, HS, NS, FS | 14 |
| Cummings 2016 | 9241 (42) | 64 | Depression, stress | 5.95 | 486 | SI | 6 |
| Fenger-Grøn 2020 | 90832 (32.6) | 40-100 | Partner Bereavement | 5 | 8823 | IS, ICH | 11 |
| Henderson 2013 | 1575 (38.22) | 77.1 | Psychosocial Distress | 6 | 603 | SI, SM | 13 |
| Kuper 2007 | 47942 women | 40.3 | Socioeconomic Status | 11.2 | 200 | IS, HS | 14 |
| Araki 2004 | 124 (32.97) | 75 | Socioeconomic state, behaviors | 3 | 25 | IS,CH | 12 |
| Arbelaez 2007 | 2312 (41.8) | 72.7 | Depression | 11 | 607 | IS | 16 |
| Bos 2008 | 1759 (39.76) | 71.2 | Depression | 15 | 291 | IS, HS, NS | 14 |
| Eurelings 2013 | 729 (40.27) | 74.12 | Apathy, depression | 2 | 55 | IS | 12 |
| Curkendall 2004 | 1495 (49.5) | 49.6 | schizophrenia | 3.3 | NM | ICH, IS, NS, TCI | 7 |
| Ohira 2001 | 311 (35.38) | 40-78 | Depression | 10.3 | 69 | IS, HS, SAH | 9 |
| Surtees 2007 | 8939 (43.33) | 41-80 | Social stress | 7.1 | 452 | FS, NFS | 13 |
| Bergh 2014 | 237879 (100) | 31-58 | Stress resilience | 23 | 3411 | IS, HS, FS | 9 |
| Blomstrand 2014 | 1460 women | 38-60 | Mental stress, socioeconomic state | 32 | 184 | IS, HS, FS, NFS | 9 |
| Everson-Rose 2014 | 3172 (47) | 45-84 | Depression, chronic stress | 8.5 | 195 | IS,HS, NS, TIA | 16 |
| Feller 2013 | 21653 (43) | 35-65 | Life satisfaction | 8 | 440 | SI | 12 |
| Hamano 2014 | 137305 (42.08) | ≥30 | Depression , interpersonal | 3 | 4718 | SI | 9 |
| Lahti 2012 | 6775 (52.36) | > 24 | Schizophrenia | 33 | 619 | SI , SM | 5 |
| Lee 2008 | 2172 (43.77) | 18-44 | Depression | 5 | 98 | SI | 10 |
| Lin 2007 | 9292 (49.68) | >18 | Bipolar disorder | 6 | 315 | SI | 10 |
| Gafarov 2013 | 870 women | 25-64 | Depression | 16 | 35 | SI | NA |
| Gafarov 2013 | 560 women | 25-64 | Depression | 16 | 35 | SI | 1 |
| Kivimaki 2007 | 3160 (55.45) | 19-65 | Job strain | 11 | 93 | IS | 1 3 |
| kornerup 2010 | 9186 (40.53 ) | 22-99 | psychological | 6-9 | 409 | HS,IS,FS,NFS | 7 |
| Nielsen 2005 | 12,096 (44.41) | 21-98 *56 | Psychological, behavioral | 14-16 | 880 | IS,HS,SAH, ICH | 7 |
| Ohlin 2004 | 13,609 (79.85) | men *46 , women * 42 | psychological | 21 | NA | CVD | 10 |
| Riahi 2023 | 6,779 (47.2) | 45-84 | Psychological, interpersonal | *8.46 | 370 | FS,NFS | 11 |
| Santosa 2021 | 118,706 | 35-70 | psychological | 13 | 2880 | NS | 17 |
| Majed 2012 | 9,601 men | 48 - 64 | psychological | 10 | 136 | IS | 13 |
| mejia-Lancheros 2014 | 7,263 (42.5) | 55- 80 | psychological | 4.8 | NA | NS | 13 |
| Nabi 2010 | 37,012 (62.7) | 20-54 | behavioral | 7 | 105 | FS, NFS,SAH, ICH,CI | 13 |
| Nilsson 2004 | 13,748 | affective disorder patients* 56.7 , osteoarthritis patients *66.6 | psychological | NA | stroke 1.8 % of affective disorder patients , 1.3 % of mania , 1.9 % of depression , 1.9 % osteoarthritis | CVD,CH | 3 |
| salaycik 2006 | 4,120 | 29-100 | psychological | 8 | 228 | HS,IS , TIA ,CVD | 8 |
| shirai 2009 | 88,175 men and women | 40- 69 | psychological | 12 | 3523 | FS,NS | 10 |
| Sumner 2015 | 49,978 | 44-62 | psychological | 20 | 271 | IS | NA |
| Schiöler 2015 | 75,236 | * 35.3 | Psychosocial, work environment | 15 | 2623 | IS | 5 |
| Tezuka 2019 | 5,936 | 40 - 79 | Anger | 3 | 312 | NM | 8 |
| Williams 2002 | 13,851 | 48-67 | Anger, Hostility | 6.4 | 228 | IS, HS | 12 |
| Xu 2022 | 135,669 | 18-65 | Psychological, occupational | 1 | NA | IS, HS | 8 |
| André-Petersson 2007 | 7,770 | 54.2 (F); 55.5 (M) | Occupational, Interpersonal | 5 | 91 | IS, HS | 7 |
| Everson 2001 | 2,303 | 52.8 | Interpersonal | 1 | 113 | IS, HS | 11 |
| Smoller 2007 | 3,369 | 50-70 | panic | 4 | NA | CVD | 20 |
| Kuan-Yi Tsai 2012 | 322,276 | 56-59 | schizophrenia | 5 | 4491 | IS, HS | 2 |
| Yan 2012 | 4,619 | ≥65 | psychosocial | 11.5 | 548 | IS | 12 |
| Glium 2003 | 5,614 | 45-72 | Education, poverty | 5 | NA | IS, HS | 8 |
| Honjo 2015 | 90,843 | 40-69 | Socioeconomic | 15.4 | NA | IS, HS | 2 |
| Iso 2002 | 73,424 | 40-79 | Psychological | 3 | 657 | IS, NS, CVD | 6 |
| Fujino 2007 | 14,568 | 40–59 | Perceived Noise Exposure | 3 | 35 | ICH | 11 |
| Jackson 2018 | 221,677 | ≥45 | Psychological | 4.7 | 2421 | MI, NS | 5 |
| May 2002 | 2,201 | 49-64 | Psychological | 14 | 130 | NS | 14 |
| Prasad 2020 | 151 | ≥18 | Acute Psychological | 1 | 151 | NS | 5 |
| Surtees 2008 | 20,627 | 76.9 | Psychological | 8.5 | 595 | NS | 14 |
| Truelsen 2003 | 12,689 | 20–98 | Self-Reported Stress | 13 | 929 | NS | 11 |
| Lawrence 1996 | 556 | 60-70 | Occupational | 6 | 44 | NS, CVD | 5 |
| Honjo 2008 | 20,543 | 40 -59 | Socioeconomic | 12 | 902 | NS, ICH | 11 |
| mcFadden 2009 | 22,488 | 39-79 | Socioeconomic | 10 | 683 | NS, CVD | 11 |
| Eng 2003 | 23,522 | 50-85 | Anger Expression | 2 | 57 | NS | 3 |
| Eriksson 2018 | 5,753 (100) | 55 | Noise exposure | NA | 336 | NS | 1 |
| Uchiyama 2005 | 1,615 (56) | 40-75 | Socioeconomic,  Work environment | 5.6 | NA | NS | 5 |
| Tsutsumi 2009 | 6,553 (48) | 18-65 | Occupational | 11 | 147 | CVD,ICH | 9 |
| Harmsen 2006 | 7,457 (100) | 47-55 | Psychological | 28 | 1019 | NS | 7 |
| Gallo 2014 | 5,313 | 18-74 | Psychological | NA | 77 | NS | 5 |
| Glover 2020 | 5,306 (36) | 21-95 | Psychological | 12 | 140 | NS | 5 |
| Harmsen 1990 | 9,998 (100) | 47-55 | Socioeconomic | 11.8 | 230 | NS | 4 |
| Metcalfe 2005 | 5,577 (100) | 34-64 | Social,  Psychological | 25 | 444 | NS | 7 |
| Nagayoshi 2014 | 13,686 (44) | 57 | Socioeconomic | 18.6 | 905 | IS, HS | 14 |
| Engström 2004 | 118,134 (44.8) | 40-89 | Psychological | 10 | 6184 | IS, HS, CVD | 3 |
| Li 2008 | 6,9625 | 40-65 | Socioeconomic | 10 | 275 | IS, HS | 5 |
| Veronesi 2010 | 5,084 | 35-74 | Psychological | 12 | 309 | IS, HS | 9 |
| Ikeda 2008 | 4,4152 | 40-69 | Socioeconomic | 10.7 | 1057 | IS, HS | 7 |
| Ohira 2009 | 1,024 | NA | Socioeconomic | 3 | 429 | IS | 5 |

*mean

IS: ischemic stroke, HS: hemorrhagic stroke, NS: non-specified stroke, FS: fatal stroke, NFS: non-fatal stroke, ICH: intracerebral hemorrhage, SI: stroke incident, SM: stroke mortality, CH: cerebral hemorrhage, SAH: subarachnoid hemorrhage, TIA: transient ischemic attacks, TCI: transient cerebral ischemia, NM: not mentioned, CVD: cerebrovascular disease, CI: cerebral infarct, MI: myocardial infarction

**Appendix T.3. Summary table of ROB assessment using the Newcastle-Ottawa Quality Assessment Scale for Case-Conrol studies.**

| Study ID | | Selection | | Comparability | | Exposure | | Quality Score | |
| --- | --- | --- | --- | --- | --- | --- | --- | --- | --- |
| Abel 1999 | 1 point | 1 point | 1 point | 1 point | 2 points | 1 point | 1 point | 0 point | 8 out of 9 |
| Jood 2017 | 1 point | 1 point | 1 point | 1 point | 2 points | 1 point | 1 point | 0 point | 8 out of 9 |
| Reddin 2022 | 1 point | 1 point | 1 point | 1 point | 2 points | 1 point | 1 point | 1 point | 9 out of 9 |
| Behymer 2025 | 1 point | 1 point | 1 point | 1 point | 2 points | 0 point | 1 point | 0 point | 7 out of 9 |
| Kuan-Yi Tsai 2012 | 1 point | 1 point | 1 point | 1 point | 2 points | 1 point | 1 point | 0 point | 8 out of 9 |
| O’Donnell 2010 | 1 point | 1 point | 1 point | 1 point | 2 points | 1 point | 1 point | 1 point | 9 out of 9 |
| Sarfo 2022 | 1 point | 1 point | 1 point | 1 point | 2 points | 1 point | 1 point | 0 point | 8 out of 9 |
| Sarfo 2018 | 1 point | 1 point | 1 point | 1 point | 2 points | 1 point | 1 point | 0 point | 8 out of 9 |
| Zaitsu 2019 | 1 point | 1 point | 1 point | 1 point | 2 points | 1 point | 1 point | 1 point | 9 out of 9 |
| Egido 2012 | 1 point | 1 point | 1 point | 1 point | 1 point | 1 point | 1 point | 1 point | 9 out of 9 |
| Riaz 2016 | 1 point | 1 point | 0 point | 1 point | 2 point | 1 point | 1 point | 0 point | 7 out of 9 |
| Khan 2023 | 1 point | 1 point | 1 point | 1 point | 2 point | 1 point | 1 point | 0 point | 8 out of 9 |

**Appendix T.4. Summary table of ROB assessment using the Newcastle-Ottawa Quality Assessment Scale for Cohort studies.**

| Study ID | Selection | | | | Comparability | Outcome | | | Quality Score |
| --- | --- | --- | --- | --- | --- | --- | --- | --- | --- |
| Albert 2006 | 1 point | 1 point | 1 point | 1 point | 2 points | 1 point | 1 point | 1 point | 9 out of 9 |
| Almas 2015 | 1 point | 1 point | 1 point | 1 point | 2 points | 1 point | 1 point | 1 point | 9 out of 9 |
| Blomstrand 2022 | 1 point | 1 point | 1 point | 1 point | 2 points | 1 point | 1 point | 1 point | 9 out of 9 |
| Cummings 2016 | 1 point | 1 point | 1 point | 1 point | 2 points | 1 point | 1 point | 1 point | 9 out of 9 |
| Fenger-Grøn 2020 | 1 point | 1 point | 1 point | 1 point | 2 points | 1 point | 0 point | 1 point | 8 out of 9 |
| Henderson 2013 | 1 point | 1 point | 1 point | 1 point | 2 points | 1 point | 1 point | 0 point | 8 out of 9 |
| Kuper 2007 | 1 point | 1 point | 0 point | 1 point | 2 points | 1 point | 1 point | 1 point | 9 out of 9 |
| Araki 2004 | 1 point | 1 point | 1 point | 1 point | 2 points | 1 point | 1 point | 1 point | 8 out of 9 |
| Arbelaez 2007 | 1 point | 1 point | 1 point | 1 point | 2 points | 1 point | 1 point | 1 point | 9 out of 9 |
| Bos 2008 | 1 point | 1 point | 1 point | 1 point | 2 points | 1 point | 1 point | 1 point | 8 out of 9 |
| Curkendall 2004 | 1 point | 1 point | 1 point | 1 point | 2 points | 1 point | 1 point | 1 point | 9 out of 9 |
| Eurelings 2013 | 1 point | 1 point | 1 point | 1 point | 2 points | 1 point | 0 point | 1 point | 9 out of 9 |
| Öhlin (2004) | 1 point | 1 point | 1 point | 1 point | 2 points | 1 point | 1 point | 1 point | 9 out of 9 |
| Surtees(2007) | 1 point | 1 point | 1 point | 1 point | 2 points | 1 point | 1 point | 0 point | 8 out of 9 |
| Bergh (2014) | 1 point | 1 point | 1 point | 1 point | 2 points | 1 point | 1 point | 1 point | 8 out of 9 |
| Blomstrand(2014) | 1 point | 1 point | 1 point | 1 point | 2 points | 1 point | 1 point | 1 point | 8 out of 9 |
| Everson-Rose (2014) | 1 point | 1 point | 1 point | 1 point | 2 points | 1 point | 1 point | 1 point | 9 out of 9 |
| Feller 2013 | 1 point | 1 point | 0 point | 1 point | 2 points | 1 point | 1 point | 1 point | 9 out of 9 |
| Hamano 2014 | 1 point | 1 point | 1 point | 1 point | 2 points | 1 point | 1 point | 1 point | 9 out of 9 |
| Lahti 2012 | 1 point | 1 point | 1 point | 1 point | 1 point | 1 point | 1 point | 1 point | 9 out of 9 |
| Lee 2008 | 1 point | 1 point | 1 point | 1 point | 2 points | 1 point | 1 point | 1 point | 8 out of 9 |
| Lin 2007 | 0 point | 1 point | 1 point | 1 point | 2 points | 0 point | 1 point | 1 point | 9 out of 9 |
| Gafarov (2013) | 1 point | 1 point | 1 point | 1 point | 1 point | 1 point | 1 point | 1 point | 8 out of 9 |
| Kivimaki 2007 | 0 point | 1 point | 1 point | 1 point | 2 points | 1 point | 1 point | 1 point | 9 out of 9 |
| kornerup 2010 | 1 point | 1 point | 1 point | 1 point | 2 points | 1 point | 1 point | 1 point | 7out of 9 |
| Kuper 2007 | 1 point | 1 point | 1 point | 1 point | 2 points | 1 point | 1 point | 1 point | 8 out of 9 |
| Nielsen 2005 | 1 point | 1 point | 1 point | 1 point | 2 points | 1 point | 1 point | 1 point | 8 out of 9 |
| Ohlin 2004 | 1 point | 1 point | 1 point | 1 point | 2 points | 1 point | 1 point | 1 point | 9 out of 9 |
| Riahi 2023 | 1 point | 1 point | 1 point | 1 point | 2 points | 1 point | 1 point | 1 point | 9 out of 9 |
| Santosa 2021 | 1 point | 1 point | 0 point | 1 point | 2 points | 1 point | 1 point | 1 point | 9 out of 9 |
| Majed 2012 | 1 point | 1 point | 0 point | 1 point | 2 points | 1 point | 1 point | 1 point | 9 out of 9 |
| mejia-Lancheros 2014 | 1 point | 1 point | 1 point | 1 point | 2 points | 1 point | 1 point | 0 point | 8 out of 9 |
| nabi 2010 | 1 point | 1 point | 1 point | 1 point | 2 points | 1 point | 1 point | 1 point | 8 out of 9 |
| Nilsson 2004 | 0 point | 1 point | 1 point | 1 point | 2 points | 1 point | 1 point | 1 point | 8 out of 9 |
| Salaycik 2006 | 1 point | 1 point | 1 point | 1 point | 2 points | 1 point | 1 point | 1 point | 9 out of 9 |
| shirai 2009 | 1 point | 1 point | 0 point | 1 point | 2 points | 1 point | 1 point | 1 point | 9 out of 9 |
| Schiöler 2015 | 0 point | 0 point | 0 point | 1 point | 1 point | 1 point | 1 point | 1 point | 9 out of 9 |
| Sumner 2015 | 0 point | 0 point | 0 point | 1 point | 1 point | 1 point | 1 point | 1 point | 9 out of 9 |
| Tezuka 2019 | 0 point | 0 point | 0 point | 1 point | 0 point | 1 point | 1 point | 1 point | 8 out of 9 |
| Tsutsumi 2011 | 0 point | 1 point | 0 point | 1 point | 2 points | 1 point | 1 point | 1 point | 5 out of 9 |
| Williams 2002 | 0 point | 0 point | 1 point | 1 point | 1 point | 1 point | 1 point | 1 point | 5 out of 9 |
| Xu 2022 | 1 point | 0 point | 0 point | 1 point | 2 points | 1 point | 1 point | 1 point | 4 out of 9 |
| André-Petersson 2007 | 0 point | 0 point | 0 point | 1 point | 2 point | 1 point | 1 point | 1 point | 7 out of 9 |
| Bergh 2014 | 1 point | 0 point | 1 point | 1 point | 0 point | 1 point | 1 point | 1 point | 6 out of 9 |
| Everson 2001 | 0 point | 0 point | 1 point | 1 point | 1 point | 1 point | 1 piont | 1 point | 7 out of 9 |
| Smoller2007 | 0 point | 0 point | 0 point | 1 point | 2 point | 1 point | 1 point | 1 point | 6 out of 9 |
| YAN1 2012 | 0 point | 0 point | 1 point | 1 point | 2 points | 1 point | 1 point | 0 point | 5 out of 9 |
| gilum2003 | 0 point | 0 point | 0 point | 1 point | 1 point | 1 point | 1 point | 0 point | 5 out of 9 |
| honjo2015 | 1 point | 1 point | 1 point | 1 point | 2 points | 1 point | 1 point | 0 point | 5 out of 9 |
| Fujino 2007 | 0 pont | 1 point | 1 point | 1 point | 2 points | 1 point | 1 point | 0 point | 6 out of 9 |
| Iso 2002 | 1 point | 1 point | 0 point | 1 point | 2 points | 1 point | 1 point | 0 point | 4 out of 9 |
| Jackson 2018 | 1 point | 1 point | 1 point | 1 point | 2 points | 1 point | 1 point | 1 point | 9 out of 9 |
| May 2002 | 0 point | 1 point | 1 point | 0 point | 2 points | 1 point | 1 point | 0 point | 7 out of 9 |
| Surtees 2008 | 0 point | 1 point | 1 point | 1 point | 2 points | 1 point | 1 point | 0 point | 7 out of 9 |
| Truelsen 2003 | 1 point | 1 point | 0 point | 1 point | 2 points | 1 point | 1 point | 1 point | 9 out of 9 |
| Lawrence 1996 | 0 point | 1 point | 1 point | 0 point | 2 points | 1 point | 1 point | 1 point | 7 out of 9 |
| Honjo, 2008 | 0 point | 1 point | 0 point | 1 point | 2 points | 1 point | 1 point | 0 point | 7 out of 9 |
| li2008 | 1 point | 1 point | 1 point | 1 point | 2 points | 1 point | 1 point | 1 point | 8 out of 9 |
| mcfadden2009 | 1 point | 1 point | 1 point | 1 point | 2 points | 1 point | 1 point | 0 point | 6 out of 9 |
| veronesi2010 | 1 point | 1 point | 1 point | 1 point | 2 points | 1 point | 1 point | 1 point | 7 out of 9 |
| Ohira 2009 | 1 point | 0 points | 1 point | 1 point | 1 point | 1 point | 0 points | 1 point | 9 out of 9 |
| Eriksson 2018 | 1 point | 1 point | 1 point | 1 point | 2 points | 1 point | 1 point | 1 point | 9 out of 9 |
| Uchiyama 2005 | 1 point | 0 point | 1 point | 1 point | 2 point | 1 point | 1 point | 1 point | 9 out of 9 |
| Tsutsumi 2009 | 1 point | 1 point | 1 point | 1 point | 2 point | 1 point | 1 point | 0 points | 5 out of 9 |
| Harmsen 2006 | 1 point | 1 point | 1 point | 1 point | 2 point | 1 point | 1 point | 1 point | 9 out of 9 |
| Gallo 2014 | 1 point | 1 point | 1 point | 1 point | 2 point | 1 point | 0 point | 0 point | 7 out of 9 |
| Glover 2020 | 1 point | 1 point | 1 point | 1 point | 2 point | 1 point | 1 point | 1 point | 9 out of 9 |
| Harmsen 1990 | 1 point | 1 point | 1 point | 1 point | 2 point | 1 point | 1 point | 1 point | 9 out of 9 |
| Metcalfe 2005 | 1 point | 1 point | 1 point | 1 point | 2 point | 1 point | 1 point | 0 point | 6 out of 9 |
| Ikeda 2008 | 1 point | 1 point | 1 point | 1 point | 2 point | 1 point | 1 point | 1 point | 9 out of 9 |
| Nagayoshi 2014 | 1 point | 1 point | 1 point | 1 point | 2 points | 1 point | 1 point | 1 point | 9 out of 9 |
| Engström 2004 | 1 point | 1 point | 1 point | 1 point | 2 points | 1 point | 1 point | 1 point | 9 out of 9 |
| Ohira (2001) | 1 point | 1 point | 1 point | 1 point | 2 points | 1 point | 1 point | 1 point | 9 out of 9 |

 **Appendix Fig.1. Psychological Factors Forest Plot after removing outliers**
**Appendix Fig.2. Subgroup Analysis forest Plot by Effect Measure Used**
**Appendix Fig.3. Gender Subgroup Analysis Forest Plot.**

 **Appendix Fig.4. Behavioral Risk Factors Forest Plot**

**Appendix. Fig.5 Psychological Risk Factors Funnel plot**

**Appendix Fig.6. Vocational Risk Factors Funnel Plot**
